# Improving the quality of care in health systems: towards better strategies

**DOI:** 10.1186/s13584-021-00448-y

**Published:** 2021-02-19

**Authors:** Jennifer Dixon

**Affiliations:** The Health Foundation, 8 Salisbury Square, London, EC4Y 8AP UK

## Abstract

Improving the quality of health care across a nation is complex and hard. Countries often rely on multiple single national level programmes to make progress. But the key is to use a framework to develop a balanced overall strategy, and evaluate the main elements continuously and over time. Achieving that requires having a critical mass of leaders who collectively can see the bigger picture now, envision a roadmap for the future to chart an intelligent course, and course correct regularly. This is a long-term agenda requiring commitment, careful stewardship, different perspectives, trust, and the building of knowledge and experience over time. It is also almost completely at odds with much current policymaking which is short term, reactive and demands hard results. Many countries are making progress. But the rapid introduction of new types of care during the COVID 19 pandemic, such as online and digital, the use of new technologies which could soon revolutionalise the way care is delivered, experienced and evaluated, and the huge pressures on spending on health care in future mean we will have to do better. Achieving system-wide quality of care requires having a critical mass of leaders who collectively can see the bigger picture now, envision a roadmap for the future to chart a balanced intelligent course. For the Israeli health system, the recent IJHPR article by Dreiher et al. will help, but it will be important, in the future, to analyse how Israel measures up on the framework outlined above. This ideally would be supplemented with a survey of key leaders for their assessment, and both would be a regular (say 5 yearly) exercise and would help inform future strategies.

## Introduction

Most governments in developed countries want to ensure their populations have accessible, high quality, affordable health care. Building blocks for achieving this objective include providing the population some form of universal coverage of comprehensive benefits, investments allowing a decent level of quality of care to be provided, and regulating care providers, in particular the medical profession.

As good quality care does not necessarily flow from these basic ingredients, most countries have developed approaches to try to ensure it. Put simply, at one end of the spectrum are those that largely seek to mitigate the worst safety risks to health, perhaps in response to significant and well publicised lapses in quality. In the middle are an extensive range of initiatives that seek to improve care each in specific high priority areas. And at the other end are countries with a comprehensive and coherent strategy comprised of multiple approaches. Many countries aspire to the latter but are in the middle part of the spectrum. The ability to design a comprehensive strategy is difficult, but the ability to deliver it is more so, given the historical context, assets and power structures within countries to make or break progress. Some countries lack the ability to make needed changes, as power over different levers is widely distributed across different parties or different levels of the system – so agreeing and implementing a national strategy is possible but considerably more difficult.

It is refreshing when occasionally, as in Dreiher et al’s report with respect to the health care system in Israel [1], there is an attempt to lay out the key approaches used in a particular health care system to improve quality and assess progress. The feat is exceptionally challenging because quality of care is a slippery multifaceted concept and difficult to measure. And initiatives cover a multitude of dimensions, from regulation to measurement to financial incentives to public reporting to patient choice and more. How individual initiatives are meant to impact on quality may not be particularly clear, still less on how they might interact with others. Some initiatives may have indirect and lagged effects and may not be seen as quality initiatives at all. While direct and significant national initiatives may be well described, how they stack up as a whole is often not.

The easier job is to compare with other countries – is one country’s set of initiatives missing anything big being tried somewhere else? Are there glaring differences in outcomes? But the more difficult task is to assess whether, taken as a whole, policies in a country represent a coherent and balanced strategy. This is a tall order for any group of national leaders to assess, be they in a ministry, university, a quality institute or professionals in the health care system itself. And yet it is important to try, and keep trying, because doing so gives the best chance to make progress.

## Concepts to consider in developing a coherent strategy

One way forward is to identify some basic concepts within a strategy, before categorising policies under them to assess balance, identify gaps, and point to where efforts should best be directed. Here I draw heavily on the work by Sutherland and Leatherman [2, 3] Molloy et al. [4], Darzi [5] and others for the NHS in England. As in other countries, in England there have been several attempts to produce an overall strategy for quality of care in the National Health Service, seen most recently in the policy High Quality Care for All led by Lord Ara Darzi, published in 2008, which attempted to put quality at the centre of policymaking [5].

The obvious first step is to be clear about what is meant by quality of care and which are the objectives to achieve in any strategy. Many countries use the Institute of Medicine’s (IOM’s) definition of six domains: safety, effectiveness, patient-centredness, timeliness, efficiency and equity (equal access for equal need) [6].

The second is to consider in a strategy the balance of three core functions in achieving high quality in any industry, as outlined in the Juran trilogy: planning; improvement; control. In the context of health care this means effective strategic planning for quality at national level; support for organisations and professionals to improve care (for example using quality improvement techniques [7]); and control mechanisms to ensure progress and mitigate risks (including regulation and inspection, and also accountability through for example management and use of metrics). These three core functions are clearly linked, and Juran thought it important not to rely on any single one. For example a country heavily relying on regulation and inspection, might drive out professional motivation to improve care, or perversely encourage behaviour which may reduce quality.

The third is to use a framework to classify and organize quality-related activity to spot potential gaps or weaknesses in a national strategy, as modified [4] from High Quality Health care for All, as shown in Table 1.

**Table 1:** A practical strategic framework for improving quality

1. SET DIRECTION AND PRIORITIES: Set clear quality priorities with desired outcomes. 2. BRING CLARITY TO QUALITY: Set standards for what high quality care looks like in key areas. 3. MEASURE AND PUBLISH QUALITY: Harness information to improve quality of care through performance and quality reporting systems that provide feedback to providers of care at systemic, institutional or individual levels; and information to users and commissioners of services for accountability and choice. 4. RECOGNISE AND REWARD QUALITY: Recognise and reward improvement in the quality of care and service through financial and non-financial recognition (eg enhanced reputation or prestige). 5. SAFEGUARD QUALITY: Use regulation to improve health care, to guarantee minimum acceptable standards and to reassure the public about quality of care. 6. BUILD CAPABILITY: Improve leadership, management, professional and institutional culture, skills and behaviours to provide quality assurance and improvement. 7. STAY AHEAD: Develop research, innovation and planning to provide progressive, high quality care.	

Source: Molloy A, Martin S, Gardner T, Leatherman S. A Clear Road Ahead. The Health Foundation, 2016

The fourth is in any strategy to pay attention to building capacity to improve quality at different levels in a country, for example at different geopolitical or administrative levels or institutions (providers or professional membership institutions for example). The aim here is to ensure that capability to improve quality and ‘ownership’ is developed at each level, and that there is synergy of activities at each level.

In a thoughtful essay comparing quality strategies in the UK and US, Ferlie and Shortell [8] describe four levels as being those operating at: individual level (such as staff education); group or team level (such as team development and pathway redesign); organisation level (such as approach to quality improvement and assurance); and larger system level (such as regulation, and public reporting of performance and outcomes).

**Fig. 1:**
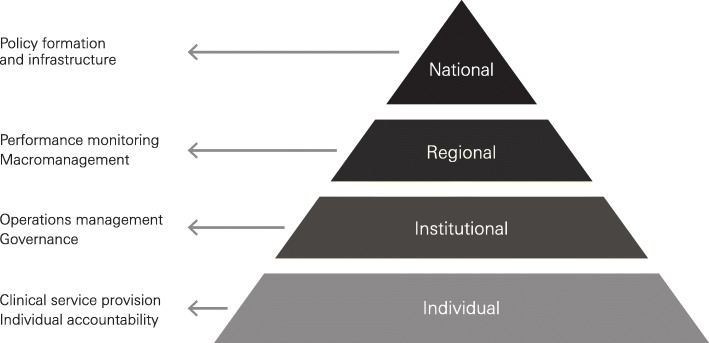
Multi-level model for building capacity for a national quality strategy. Source: Molloy A, Martin S, Gardner T, Leatherman S. A Clear Road Ahead. The Health Foundation, 2016

Molloy et al [4] have a slightly different approach to categorising the multiple levels where action is needed to improve quality, illustrated in the pyramid in Fig. 1.

In the pyramid the four levels refer broadly to the following:

- national – policy formulation, resourcing, infrastructure and accountability to the public
- regional/local – translating national policy into the local context, macro-management and monitoring
- institutional – good governance, competent operational management and continuous quality improvement
- individual – this is the level of encounter between patients and health professionals where the key attributes of quality must be actualised through individual behaviours.

The fifth concept (adapted from Leatherman and Sutherland) is to consider initiatives according to who or what is their intended target – people individually or collectively involved in health care delivery, or organisations at national, regional and local level that form part of the health system. Given the myriad of initiatives, some only indirectly targeting quality, it is important for any strategy to define the scope of what might be included. For example, to what extent is, say, criteria for capital investment important for improving quality, or initiatives to improve coding of data used to measure quality?

## Putting it all together

In 2016 a comprehensive independent assessment of the main approaches to improve the quality of care in the NHS in England using these five concepts was published [4]. In brief, the findings revealed a very large number of national initiatives directly to improve quality (179 announced by the government alone over the previous 4 years), many in response to hospital-based lapses in care and heavily focused on patient safety (70% of initiatives). Given this, many were skewed towards Juran’s ‘control’ (regulation and reporting metrics, such as the introduction of national chief inspectors of care and a publicly reported system for rating the quality of primary, social and hospital care) rather than ‘improvement’ (supporting clinicians for example by developing quality improvement skills). The government initiatives, and many more (for example coming from national public agencies), were aimed at all levels of the pyramid shown in Fig. 1.

More initiatives were targeted on ‘system’ and patients and the public, and far fewer on the clinical staff delivering care, yet an accompanying survey of national leaders showed workforce-focused initiatives were thought to be among the ‘best bets’ for protecting and enhancing quality. A significant step forward was the introduction of relicensure of physicians (known in the UK as ‘revalidation’) every 5 years by the General Medical Council in 2012 (https://www.gmc-uk.org/registration-and-licensing/managing-your-registration/revalidation), linked to a formal annual appraisal.

The evidence supporting the design and introduction of initiatives was frequently weak or absent, and it was also not always clear the extent of consensus among leaders on these when evidence ran short. There were examples however when a conscious effort had been made by government to press the rationale for, gather and challenge key stakeholder views to find a way forward when evidence was incomplete, for example in the work on whether to introduce a controversial national system of ratings of providers in health and social care [9].

## Implementation and evaluation

Clearly whatever the ultimately designed strategy, what is implementable and when, involves a complex set of choices, depending in part on context (what is possible) as well as a selection of priorities. But given the dynamic interplay between different elements of a quality strategy when being implemented, and the length of time to show impact, monitoring progress and formal evaluation of impact is key. The assessment of quality initiatives in the NHS in England showed that accountability for their implementation (as opposed to accountability for other managerial and clinical must-dos) and monitoring was not strong. Many initiatives were introduced at different times and overlapped in what has been described elsewhere as a ‘policy thicket’ [10]. Overall, in one-third of initiatives implementation was found to be monitored although only the biggest, high profile and national initiatives were both monitored and formally evaluated. The long lag time in implementation meant often new initiatives were overlain on older ones before their effect could be seen. The fundamental point here, too often repeated, is the importance of monitoring implementation, the need to have a stronger system of independent evaluation, and to design a system where enough people see this information to modify the course of implementation or the overall strategy.

## Building a long-term commitment

Clearly achieving high quality care is highly complex, and a moving target. Factors that will help progress include clarity and balance in elements of a multi-level strategy, wise choice of do-able initiatives, investment, competent and well-monitored implementation, solid evaluation and patience in the pace of progress. As Ferlie and Shortel noted in their analysis of quality strategies in the UK and US ‘efforts to date relied on ‘relatively narrow single-level programmatic strategies’ and that ‘well intentioned efforts will fail to realise their potential unless both policymakers and practitioners consider and implement a more comprehensive multi-level approach to change’ [8].

Achieving that surely requires having a critical mass of leaders who collectively can see the bigger picture now, envision a roadmap for the future to chart a balanced intelligent course, and course correct regularly. Dreiher et al’s contribution will help, but it will be important, in the future, to analyse how Israel measures up on a systematic framework such as the one outlined above. This ideally would be supplemented with a survey of key leaders for their assessment, and both would be a regular (say 5 yearly) exercise and would help inform future strategies.

It is worth emphasising that as quality of care will never be fully measurable, particularly the more intangible human aspects of care like empathy, kindness and understanding, any strategy must also nurture core professional values to do what is in the best interests of their patients. This is a long-term agenda in of itself requiring commitment, careful stewardship, different perspectives, trust, and the building of knowledge and experience over time. It is also almost completely at odds with much current policymaking which is short term, reactive and demands hard results.

Countries have and continue to make huge progress, as clearly demonstrated by Dreiher et al in Israel [1], in England [2, 4] and internationally by OECD [11] among others. But the question on the table now is can we move faster? The agenda is more urgent given the rapid introduction of new types of care during the COVID 19 pandemic, such as online and digital, the crowding on the horizon of new technologies which could soon revolutionalise the way care is delivered, experienced and evaluated, the huge pressures on spending on health care by governments, employers and individuals, and the changing burden of risk and ill health in the population. We will have to do better.

## Short bio

Dr. Jennifer Dixon is the chief executive of The Health Foundation, an independent philanthropic foundation based in London. She originally trained in medicine, is a specialist in public health and health policy research, and has published widely.

## Data Availability

not applicable.
